# The Use of Integra Dermal Regeneration Template in Exposed Bone Reconstruction: A Case Report with Systematic Literature Review

**DOI:** 10.3390/jcm14092971

**Published:** 2025-04-25

**Authors:** Marko S. Jović, Branko J. Suđecki, Ivan Ljubiša Radosavljević, Milan D. Jovanović, Milan T. Stojičić, Jelena D. Isaković Subotić, Nataša D. Nejković, Zorka M. Inić, Marina M. Stojanović, Jelena V. Jeremić

**Affiliations:** 1 Clinic for Burns, Plastic and Reconstructive Surgery, University Clinical Center of Serbia, 11000 Belgrade, Serbia; 2 Faculty of Medicine, University of Belgrade, 11000 Belgrade, Serbia; 3 Center for Anesthesiology, University Clinical Center of Serbia, 11000 Belgrade, Serbia; 4 Institute for Oncology and Radiology of Serbia, 11000 Belgrade, Serbia

**Keywords:** Integra, burns, bone devoid of periosteum, burring, dermal regeneration template, bone, scalp, skin grafting

## Abstract

**Background/Objectives**: Integra Dermal Regeneration Template (IDRT) has emerged as a viable reconstructive option in exposed avascular structures, such as exposed bone devoid of periosteum. This systematic review aimed at examining success rates by comparing different wound types and their characteristics, as well as the surgical methods involved. **Methods**: A systematic review was conducted to identify studies using IDRT in the reconstruction of defects with exposed bone devoid of periosteum. Primary outcomes of interest were IDRT and skin graft success rates, followed by patient and wound characteristics, and different surgical methods used. The results were accompanied by an illustrative case report of IDRT-based hand reconstruction after a deep burn injury. **Results**: The review included 40 studies, with a total of 202 individual defects. The primary indication for IDRT-based reconstruction was post-oncologic defects in the elderly population. Although surgeons mostly used burring/fenestration as a bone preparation method prior to IDRT placement, decorticated bones showed faster grafting time (23.8 vs. 27.9 days). The average success rate of IDRT was 87.54% (±25.9), with an excellent IDRT take rate (100%) observed in more than 50% of cases. In the majority of cases (95.5%), the skin graft acceptance rate was deemed to be higher than 95%, with an average graft take of 98.8%. **Conclusions**: The results of this review support the use of IDRT in managing complex defects involving exposed bone, offering fast coverage with good functional restoration, without any donor site morbidity. Additionally, bone preparation methods also play an important role in IDRT-based reconstruction by shortening the grafting time.

## 1. Introduction

The management of defects involving denuded vital structures such as exposed bone, tendon, or nerves remains a significant reconstructive challenge. Common methods include direct closure, skin grafting, local or distant flaps, microvascular free tissue transfer, or allowing spontaneous wound healing by secondary intention [1]. Choosing the right method generally depends on the depth of the wound, the availability of donor tissue, and patient-related factors such as age, general physical condition, comorbidities, expectations, and their ability to take care of the reconstructed defect [2]. Traditionally, these types of wounds have been managed using flap surgery or microvascular free-tissue transfer. While these methods offer full-thickness defect reconstruction, they often require more complex surgical or microsurgical procedures. Furthermore, each potential failure limits future reconstructive options [3,4].

Since Burke et al. published their study on the use of artificial dermal substitutes in covering full-thickness burn injuries in 1981, Integra Dermal Regeneration Template (IDRT; Integra Life Sciences Corp., Plainsboro, NJ, USA) has been used in various reconstructive scenarios, starting with major burn surgery [1,5,6,7,8,9,10]. IDRT and other acellular dermal substitutes have also been found to be a good and reliable option in the treatment of complex traumatic soft-tissue defects [11,12]. Several studies have described using IDRT to cover partially avascular wounds [1], as well as studies involving IDRT in diabetic foot reconstruction [8,13]. The use of negative pressure wound therapy (NPWT) in combination with IDRT has also been described, improving IDRT success rate and vascularization time, even when used on complex wounds [14,15,16,17].

Although several literature reviews have been published regarding the use of IDRT on different wound types [18,19,20], no reviews have specifically focused on its use on exposed bone devoid of periosteum. In line with this, we present an illustrative case report of a patient who underwent IDRT-based reconstruction of complex hand defects due to a burn injury, accompanied by a comprehensive systematic literature review of articles reporting the use of IDRT in managing exposed bone defects of different backgrounds.

## 2. Materials and Methods

**Figure 1:**
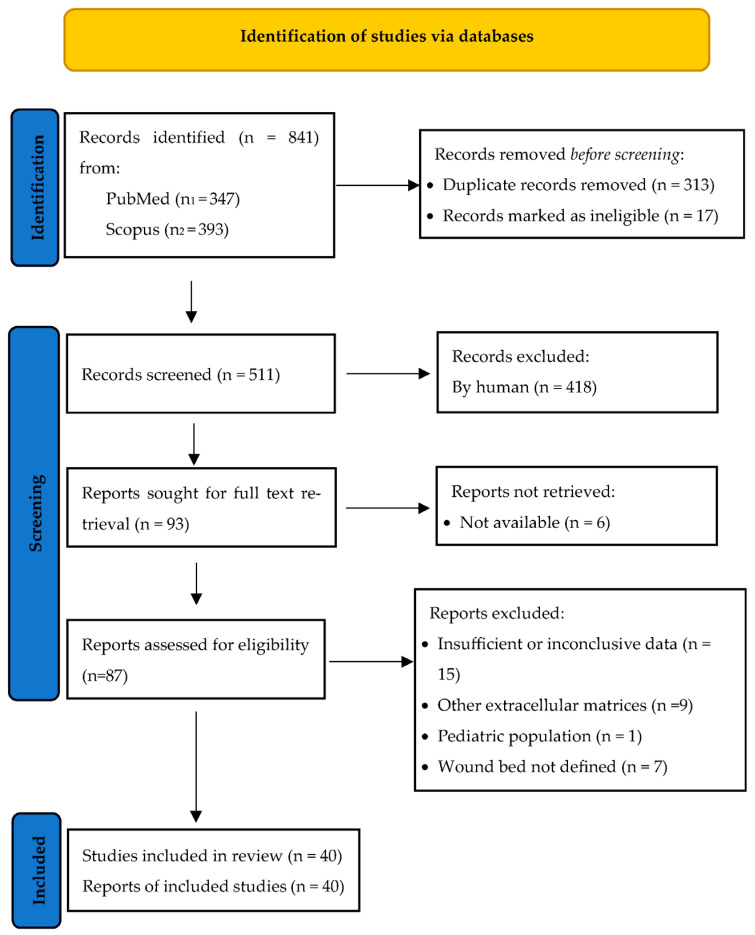
PRISMA (Preferred Reporting Items for Systematic Reviews and Meta-Analyses) flowchart.

This review followed the Preferred Reporting Items for Systematic Reviews and Meta-Analyses (PRISMA) (Figure 1) and has been registered with PROSPERO (CRD42023426130).

### 2.1. Search Strategy

A systematic literature review was conducted using the following databases: PubMed (National Center for Biotechnology Information), Scopus (Elsevier), and Web of Science (Clarivate). The search was conducted using the keywords “Integra”, “DRT”, “Dermal Regeneration Template”, and “Bone”, entered singularly or in combination to refine the research topic. Data collection and extraction lasted from 1 December 2023, to 28 February 2024, with the final electronic search performed on 18 March 2025. Reference lists of selected articles and related reviews were also hand-searched for possible additional matches. Conference proceedings were not included in the search. All study designs were considered for inclusion.

### 2.2. Study Selection

Search results were combined and deduplicated using Rayyan AI tool (Qatar Computing Research Institute, HBKU, Doha, Qatar), with human full proofing of all discrepancies. Two independent researchers (B.S. and I.R.) then conducted a two-stage assessment of the remaining articles. In the first stage, publications not matching the inclusion criteria by title and abstract were excluded. Disagreements were resolved by a third independent researcher (M.S.J.). Any discrepancies in the literature interpretation were discussed and resolved in a panel discussion. Based on each reviewed section, the resulting search strategies were further refined or expanded, as appropriate. Inquiries to the corresponding authors regarding restricted-access articles were made, and positive responses were included in the final review. In the second stage, available full-text versions of the remaining articles were further analyzed, and those meeting the inclusion criteria were selected for data extraction. The inclusion and exclusion criteria are summarized in Table 1. Long-term patient follow-up was not within the review’s scope.

### 2.3. Data Extraction

Data were extracted using a piloted data extraction sheet (Microsoft Excel) developed for this review. The variables of interest are summarized in Table 2. If precise data on the defect size or success rates were unavailable, supplementary article photographs were used by all three researchers to assess the data on a case-by-case basis, and a mean value was determined. All orbital exenterations in this review were done in a standardized way, and for assessing the size of these defects, supplementary data or the size of applied Integra^©^ DRT was used, as appropriate. Success rates described as “good”, “excellent”, and “satisfactory” were interpreted as 100% if no supplementary photographs were available. Descriptive statistical analysis was performed using IBM SPSS Statistics, Version 25. Due to the limitations of available data, quantitative analysis beyond basic descriptive reporting was not conducted.

## 3. Case Report

**Figure 2:**
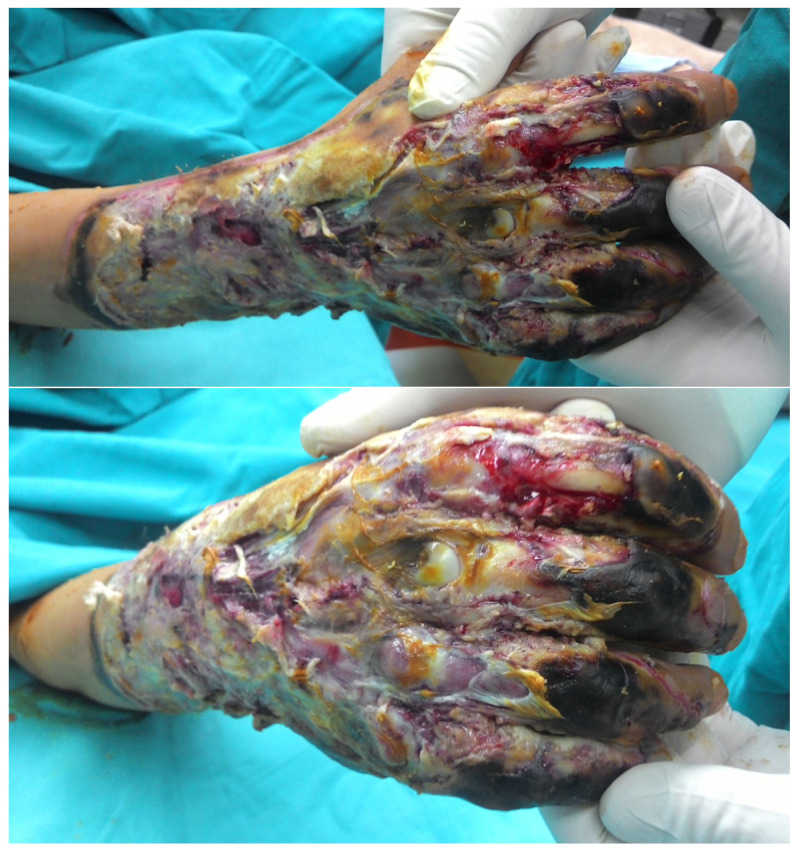
Patient presentation upon admission.

We present the case of a 24-year-old male admitted to our Burns Ward with deep fourth-degree burns involving the right forearm and hand, sustained four days prior in contact with an electrical heater. The patient was initially treated at a regional hospital and then referred to our facility. Detailed inspection upon admission revealed deep third- and fourth-degree burns on the posterior and medial forearm, as well as the entire dorsal hand surface, with exposed dorsal phalangeal bones of the II and III finger and the III metacarpophalangeal (MCP) joint (Figure 2).

**Figure 3:**
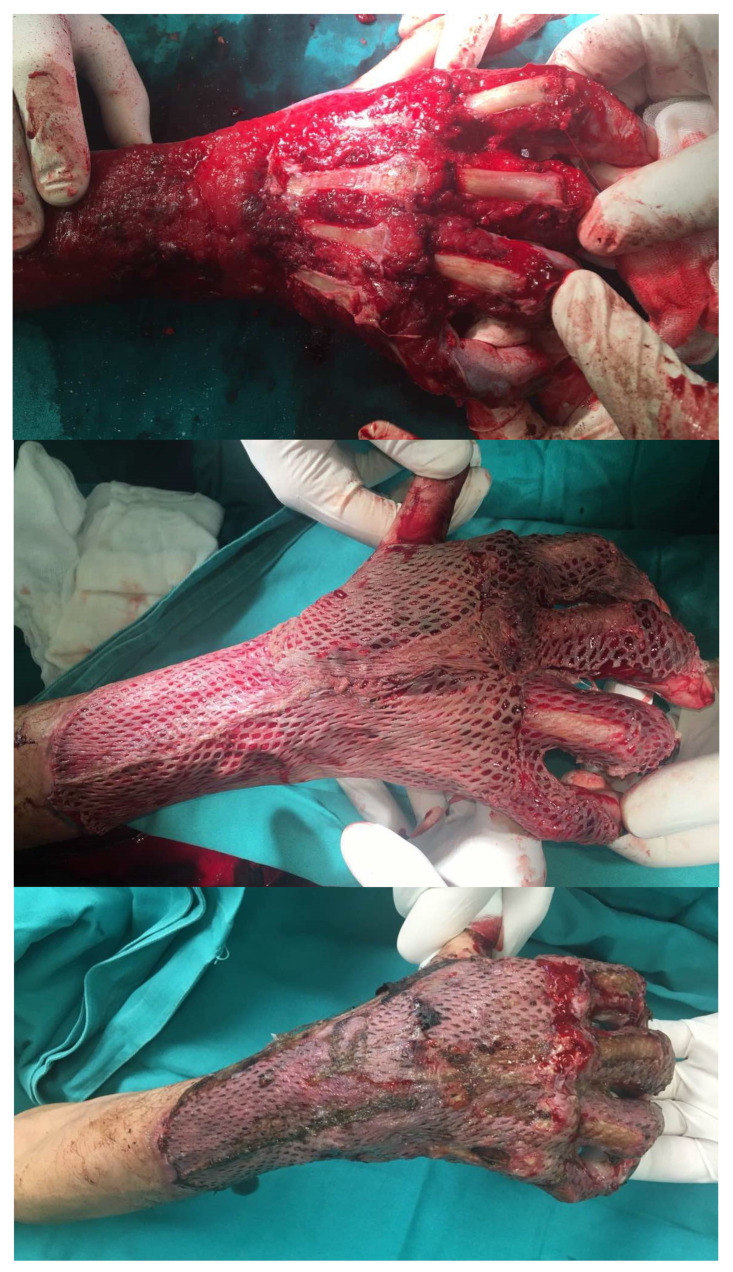
Partial osteotomy of exposed bones with skin grafting 28 days after admission. Postoperative results 7 days after grafting showing STSG failure over exposed bones.

Over the course of 11 days, regular dressing changes with multiple surgical debridements of devitalized tissue were performed, leaving exposed segments of the ulna, partial exposure of the radiocarpal joint, exposed dorsal part of II-III MCP joint and II-V proximal interphalangeal (PIP) joints, dorsal surfaces of II-V proximal phalanges, and III-V metacarpal bones. Following the final debridement session, negative pressure wound therapy (V.A.C., 3M, Solventum, Saint Paul, MN, USA) was applied to promote granulation tissue formation, with dressing changes performed every 3–5 days. On the 28th day of hospitalization, the patient underwent curettage of hypergranulation tissue with partial osteotomy of exposed bone surfaces. The wounds were covered using a split-thickness skin graft (STSG) harvested from the right thigh. The remaining exposed bone regions involved III-V metacarpal bones and the dorsal surface of II-V proximal phalanges, totaling 27.3 cm^2^ (Figure 3).

**Figure 4:**
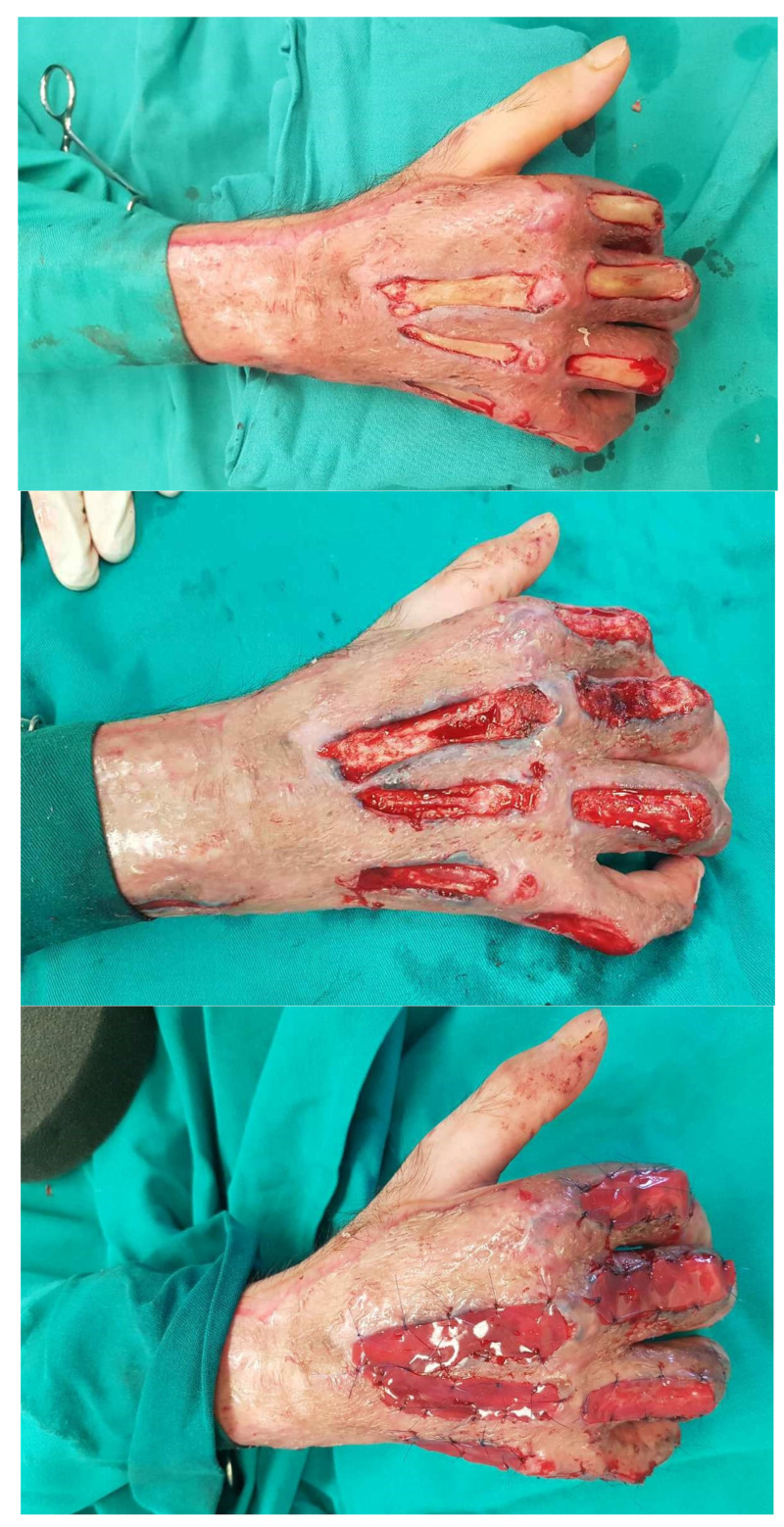
The remaining exposed bone defects, followed by a partial osteotomy and IDRT placement.

After continuous changes, 34 days later, decortication of all exposed bones regions was performed, followed by the application of pie-crusted Integra Dermal Regeneration Template (IDRT). The bolstering was done using negative pressure wound therapy (V.A.C., 3M), with dressing changes performed on the third day (Figure 4).

After 21 days, sufficient neodermal wound bed establishment allowed for silicone layer removal and skin grafting using STSG harvested from the right arm.

**Figure 5:**
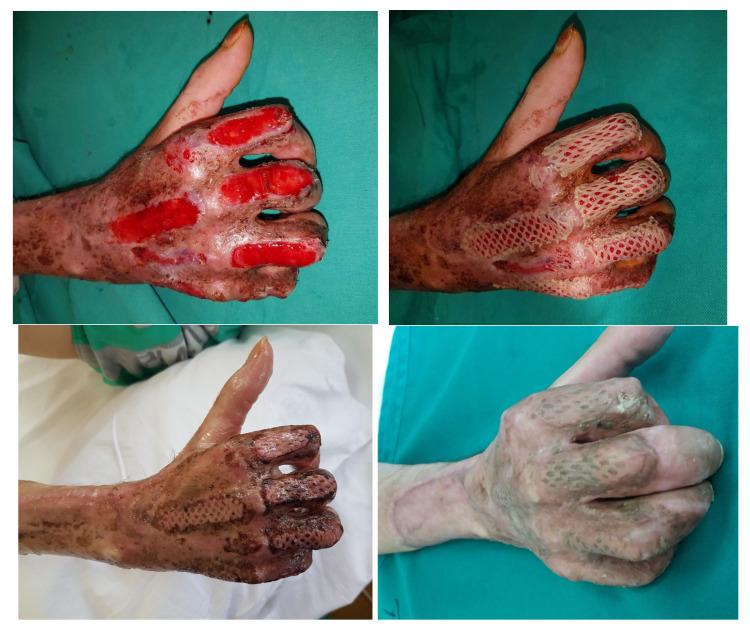
Neodremal formation after silicone layer removal on the 21st postoperative day, with immediate split-thickness skin grafting. Postoperative results five days later, and on a 3-month follow up with no skin breakage noted.

The standard STSG bolstering technique was used (vaseline gauze, ethacridine lactate solution 0.1% dressing and bandages). Both the IDRT and STSG acceptance rates were 100%, with no reported perioperative complications. The patient was later referred to a local health center for intensive hand therapy. At the three-month follow-up visit, no skin breakage was noted (Figure 5).

## 4. Results

### 4.1. Literature Review

A total of 840 records were obtained through a database literature search, and 1 article was added manually after a reference search. After deduplication and removal of ineligible articles, 511 records were chosen for abstract screening. Upon initial exclusion of 418 papers based on their title and abstract content, the remaining 93 reports were sought for full-text retrieval, of which 87 were successfully assessed for eligibility. The final systematic review included 40 studies with a total of 202 defects that met the inclusion criteria [7,8,9,10,16,21,22,23,24,25,26,27,28,29,30,31,32,33,34,35,36,37,38,39,40,41,42,43,44,45,46,47,48,49,50,51,52,53,54,55]. The reviewed articles included 20 case reports, 4 case series, 5 retrospective cohort studies, and 11 retrospective chart reviews.

### 4.2. Patient Demographics and Defect Characteristics

The median age of the sampled patients was 82 years (range 19–101), with 82.2% of patients being in the 60+ age group. Based on the data, the primary indications for IDRT-based reconstruction were post-oncologic defects (76.7%), followed by trauma injuries (9.4%), chronic wounds (8.4%), and burns (5.4%). In younger patients, the most common defect origin was burn injuries. Defect sizes ranged from 1 to 256 cm^2^ (median 32.5 cm^2^), with the majority of defects being <50 cm^2^. In 70.3% of cases, the defects involved the scalp, followed by the foot (9.9%), orbital socket (7.9%), finger (5%), leg (4%), arm (1.5%), and hand region (1.5%) (Table 3).

### 4.3. Surgical Methods and Reconstruction Protocols

In 80.2% of cases, defects were reconstructed using a two-stage approach, with most surgeons opting for split thickness skin grafting (97.5%). Before IDRT application, 87.6% of defects underwent bone preparation, most commonly bone burring and/or fenestration (70.1%). The median grafting time was 30 days (range 6–67 days), with 50% of the cases being grafted in 28–35 days. Decorticated bones had a faster average grafting time compared to burried/fenestrated bones (23.8 vs. 27.9 days). Most surgeons (84.7%) used IDRT with the silicone layer intact with standard bolstering, while negative pressure wound therapy (NPWT) was used in only 17.8% of cases (Table 4).

### 4.4. Outcomes and Complications

The average success rate of IDRT was 87.54% (±25.9%), with an excellent IDRT take rate (100%) observed in more than 50% of cases and >87% in 89.1% of cases. In most cases (95.5%) skin graft acceptance rate was deemed to be higher than 95%, with an average graft take of 98.8%. Complications were reported in 17.8% of cases, with the most common being infection (47.2%) (Table 5).

## 5. Discussion

Literature data on IDRT as a reconstructive option are plentiful. Certain studies highlight its possible role in treating avascular wound beds, both in animal models and in human studies [1,56]. Initially used for temporary coverage of large burn defects [6,57,58,59], IDRT has over time found use in the treatment of a variety of wounds [60,61]. The most common reason for IDRT use in this review was post-oncologic scalp defects in older patients [7,25,29,31,36,37,38,39,40,41,43,45,46,47,48,51].

The most traumatic injuries involving exposed bone in this review resulted from complex extremity soft-tissue injuries [9,10,22,24,27]. Using IDRT in these circumstances offers a viable coverage alternative with no donor site morbidity, possible outpatient treatment, and sufficient aesthetic and functional results. Burn injuries involving the periosteum predominantly involved the scalp and pretibial regions. These areas are commonly exposed to thermal agents and have very little soft tissue coverage, making them susceptible to bone exposure [32,42,50,54]. Although chronic wounds mostly involved the foot [23,39,44], Vegesack et al. described the use of IDRT in the rapid coverage of a large scalp defect originating from giant-cell arteritis-related skin necrosis, where impaired tissue perfusion and immunosuppressive therapy greatly interfered with wound healing [44]. Moberly et al. used IDRT to manage an unusual complication of rhytidectomy, reporting satisfactory results even in aesthetic surgery patients [23].

Based on the reports included, chronic wounds had the highest average IDRT success rate (97.9%). This could be due to the destructive nature of trauma and burn wounds, leading to larger defects with excessive tissue loss and poor wound beds. Additionally, the invasive nature of malignant tumors often requires wide and radical excisions, leading to large and complex defects. However, these findings should be interpreted with caution due to the small number of studies describing IDRT use in chronic wounds retrieved from this review.

Defect sizes in the head and neck area ranged from 2.5 to 256 cm^2^, with 88% of cases having >87% IDRT success rate and 40.8% reporting a 100% IDRT take rate. Three authors described the use of IDRT in post-exenteration orbital socket reconstruction, reporting excellent success rates (100%) [33,34,35]. Although the periosteum was not penetrated by the tumor, the exenteration technique described involved the complete removal of the periosteal sac. Using IDRT prevents mobilization of the eyebrow and midface region, which can lead to aesthetically unsatisfactory scarring results. Additionally, IDRT strengthens the chances of obtaining an open bowl-shaped socket, facilitating good future prothesis retention [34,35].

Trauma defects had the smallest median surface area (18.62 cm^2^). This suggests that wounds with exposed bone and compromised wound beds and smaller or medium-sized defects would be more appealing to reconstruct, considering the size of IDRT needed and the potential complications such as infection and primary IDRT failure. This finding is consistent with our case.

In this review, 87.6% of defects went through some form of bone preparation prior to IDRT placement, while the remainder had IDRT directly placed on the intact bone. This approach was mostly employed in post-oncologic scalp resections smaller than 50 cm^2^. The average IDRT success rate in burred/fenestrated vs. decorticated bones was 84.1% and 96.2%, respectively. Decorticated bones had a faster average grafting time (23.8 vs. 27.9 days), despite burring being employed in smaller defects (48.8 cm^2^ vs. 81.7 cm^2^). This could be due to the fact that while bone burring/fenestration allows for rapid and selective abrasion [62], decortication offers more even debridement level and vascular tissue distribution, offering faster IDRT integration, making it a more appropriate form of bone preparation where possible. Although these findings are consistent with our case, this topic requires more research. In both cases, the average skin graft take rate was > 98%.

Meshed/pie-crusted IDRT was almost always used with some sort of bone preparation, and often with NPWT bolstering. In only one case, the surgeon placed meshed IDRT on a native bone [28]. Although it did not affect its take rates, meshed IDRT was skin-grafted almost twice as fast compared to intact IDRT (16 vs. 30 days). Employing NPWT can reduce the risk of infection by effectively removing wound secretions and inflammatory factors, promoting fibroblast proliferation and maintaining the integrity of the neovascular network structure [63].

NPWT bolstering has been used in only 17.8% of cases, mostly in larger defects (mean 61.9 cm^2^), and in most cases (92%) with some form of bone preparation. This bolstering technique was used on native bone in only two cases [28,53]. IDRT used with NPWT bolstering revascularized significantly faster compared with the standard bolstering (average 19.3 days vs. 27.8 days, respectively). Both groups had satisfactory IDRT success rates (>87% and >95%, respectively).

In 80.1% of cases, a two-stage reconstruction approach was employed, with almost all surgeons (98.7%) using split-thickness skin graft (STSG) as the second-stage choice. The staged approach allows for better template revascularization control, ensuring better grafting success, but it also necessitates a second procedure. The timing of the second stage largely depended on wound-bed vascularization. In 51.5% of cases, grafting was performed after >30 days. This is due to bones devoid of periosteum being poorly vascularized wound beds, requiring more vascularization time. In addition, full revascularization is usually assessed with a change in IDRT color, which can be subjective on a case-by-case basis. In only a handful of cases, grafting was performed after >35 days [7,16,38,40,41]. Some studies have documented the successful use of Integra Single Layer DRT in defect reconstruction in combination with skin grafting, reporting excellent outcomes; however, they are out of the scope of this review [53,64].

Native-bone wound beds were grafted within the four-week period, while most cases where bone was burried/fenestrated required >30 days for grafting. In patients with meshed/pie-crusted IDRT, grafting was performed faster (16.8 vs. 28.1 days, respectively), despite these patients having larger defects (80.5 cm^2^ vs. 48.8 cm^2^, respectively). This could be because breaking the integrity of the silicone layer facilitates blood and seroma evacuation, thus strengthening the template’s adherence to the bone surface, especially when combined with NPWT bolstering.

The average STSG success rate was 98.8%, with 66.8% of cases achieving a 100% take rate. Three authors reported using full-thickness skin grafts (FTSG) as a definite coverage option for smaller hand and mandible defects, all reporting an excellent graft take [24,31,55]. One author used preserved subcutaneous vascular network skin grafting (PSVNSG) [55], which preserves the vascular network within the areolar tissue between the dermis and fat layer [55,65]. Although offering superior results compared to other skin graft types, this method requires more detailed preparation of the wound bed, making it more suitable for small defects involving important functional regions such as the fingertips, especially the thumb. Three authors reported a single-stage approach in several patients due to rapid wound epithelization [5,7,29].

Complications were reported in 17.8% of cases, with infection (47.2%) being the most common issue [29,40,43,44]. In nine of the cases described, the infection led to complete IDRT failure, while the remainder had an IDRT success rate > 75%. All reported cases involved the scalp region, and 94.1% used intact IDRT with standard bolstering. Koenen et al. observed infection in only one patient, salvaging the template by removing the silicone layer and applying NPWT for the next four weeks [40]. Vegesack et al. resolved infection with partial IDRT failure by performing debridement with simultaneous single-layer IDRT and STSG placement, while the remaining defect was grafted in a separate procedure [44]. The complication occurrence in this patient can be explained by poor wound perfusion due to giant-cell arteritis combined with obligatory immunosuppressive therapy.

Two authors have reported hematoma formation under IDRT while treating scalp defects [40,51]. In both cases, IDRT was applied intact using the standard bolstering technique, with IDRT success rates in both cases being >90%. A handful of cases described local tumor recurrence, ethmoid fistula formation, and ectropion, unrelated to IDRT use [7,35,37]. Primary IDRT failure was reported in 6 cases, while neodermal loss with/without subsequent skin breakage was reported in 8 cases, with all defects having heterogenous etiology, localization, and reconstructive approach [7,10,16,25,26,30,41,42,48,50,53].

### Limitations

The reports included in this review had various designs, mostly as case reports and case series, which could limit the generalization of findings across different types of exposed bone defects treated using IDRT. Since most of the patients included were older, the findings may not fully apply to younger patients. Assessment of defect sizes using complementary photographs could have impacted the final findings and their applicability in clinical practice. Therefore, future research should standardize preoperative defect measurement and reporting. Also, the different surgical techniques used across the studies make it harder to compare results. Some authors reported outcomes in descriptive terms rather than percentages, which could potentially underestimate or overestimate the true effectiveness of IDRT. Additionally, there is a risk of publication bias, where studies with positive outcomes are more likely to be published, especially regarding case reports.

## 6. Conclusions

Integra Dermal Regeneration Template (IDRT) is a reliable reconstructive tool for exposed bone defects across various clinical scenarios, particularly in post-oncologic scalp reconstruction in older patients. With high success rates and no donor site morbidity, IDRT’s two-stage approach effectively facilitates neovascularization and wound closure. In addition, bone preparation also plays an important part, providing good vascular support in the early stages of the template’s incorporation. Despite encountering complications such as infection, IDRT offers significant benefits in achieving satisfactory functional and aesthetic outcomes.

## Figures and Tables

**Table 1:** Summarized article inclusion and exclusion criteria.

Inclusion Criteria	Exclusion Criteria
Full length, peer-reviewed, English-language articles	Non-English articles
Adult population	Pediatric population
Use of Integra^©^ Dermal Regeneration Template in reconstruction of defects involving exposed bone devoid of periosteum	Other manufacturer dermal regeneration templates used in reconstruction of defects involving exposed bone devoid of periosteum
	Previous systematic or literary reviews and meta-analysis, clinical guidelines, editorials and commentaries, in vitro and animal studies
	Integra^©^ Dermal Regeneration Template used for reconstruction of the defects involving exposed bone with the periosteum intact, other soft tissue defects, or mixed tissue defects (soft tissue, tendon and bone) with the size of exposed bone not being properly defined
	Studies with insufficient or inconclusive data regarding the defect or performed operative procedures No acellular dermal matrices were used in reconstruction
	Integra^©^ Bilayer Wound Matrix and other Integra^©^ products

**Table 2:** Variables of interest for this systematic review.

Patient and Wound Characteristics	Surgical Methods	Outcomes
Patient age	Bone preparation	IDRT success rate
Localization	IDRT meshing	Skin graft success rate
Etiology	Bolstering technique	Complications
Size	Grafting time	
	Type of skin graft used	

**Table 3:** Demographics and defect characteristics of patients that underwent IDRT-based reconstruction. All values are reported as mean ± SD where possible. In cases where multiple patients are included in the study, the number of patients experiencing the descriptor is included in parentheses.

References	No. of Cases	Avg. Patient Age	Defect Etiology	Defect Localization	Defect Size (cm^2^)
Smock et al. (2010) [21]	1	44	Oncologic	Finger	1
Iino et al. (2017) [55]	2	61.5	Oncologic	Finger	1.5
Weigert et al. (2011) [22]	3	79	Trauma	Finger	2 ± 1
Moberly et al. (2014) [23]	1	65	Chronic wound	Scalp	2.5
Choughri et al. (2020) [9]	4	35 ± 19.23	Trauma	Arm (1), finger (3)	3.25 ± 0.96
Reynolds et al. (2018) [24]	3	55 ± 1.73	Oncologic (2), Trauma (1)	Hand	3.67 ± 2.08
Prystowsky et al. (2001) [25]	1	74	Oncologic	Scalp	6.7
Lee et al. (2008) [26]	2	27 ± 2.83	Burns	Finger (1), Foot (1)	7 ± 1.41
Ribeiro et al. (2018) [54]	1	41	Burns	Leg	8.5
Pu et al. (2009) [27]	1	52	Trauma	Foot	10
Pagnini et al. (2009) [28]	1	31	Chronic wound	Arm	12
Lee et al. (2024) [53]	1	66	Trauma	Leg	18.62
Mogedas-Vegara et al. (2021) [29]	66	83.3	Oncologic	Scalp	22.03 ± 2.84
Silverstein et al. (2006) [30]	1	65	Chronic wound	Foot	25
Singh et al. (2016) [31]	1	78	Oncologic	Mandible	27
Yeong et al. (2006) [32]	1	44	Burns	Scalp	30
Ameloot et al. (2019) [33]	1	42	Oncologic	Orbital socket	32
Ozgonul et al. (2018) [34]	5	74 ± 15.33	Oncologic	Orbital socket	32
Ćertić et al. (2023) [52]	1	46	Burns	Leg	35
Monjanel et al. (2019) [35]	10	71.3 ± 14.97	Oncologic	Orbital socket	35.23 ± 8.70
Patel et al. (2022) [36]	1	61	Oncologic	Scalp	42
Gonyon et al. (2003) [37]	2	43.5 ± 33.23	Chronic wound (1), Oncologic (1)	Scalp	42.37 ±53.22
Helgeson et al. (2007) [10]	5	Not reported	Trauma	Foot (4), Leg (1)	45 ± 11.18
Sartore et al. (2015) [38]	1	35	Oncologic	Scalp	52
Schiavon et al. (2016) [39]	17	73	Oncologic (13), Trauma (3), Chronic Wound (1)	Scalp	56.18 ± 0.53
Koenen et al. (2008) [40]	13	79.46 ± 10.30	Oncologic	Scalp	62.08 ± 36.06
Nakhi et al. (2018) [8]	5	59.4 ± 11.01	Chronic wound	Foot	71 ± 35.43
Bernstein et al. (2020) [41]	14	90.29 ± 4.81	Oncologic	Scalp	74
Yeong et al. (2012) [42]	4	67.5 ± 14.48	Burns	Scalp (2), Leg (1), Foot (1)	77.5 ± 82.06
Fraccalvieri et al. (2012) [16]	7	73.86 ± 9.75	Chronic wound	Foot	80 ± 84
Komorowska-Timek et al. (2005) [7]	4	71.25 ± 2.50	Oncologic	Scalp	92.25 ± 43.21
Vithlani et al. (2017) [43]	1	87	Oncologic	Scalp	96.7
Vegesack et al. (2015) [44]	1	86	Skin necrosis	Scalp	100
Souéid et al. (2013) [45]	5	85 ± 6.78	Oncologic	Scalp (4), Arm (1)	126 ± 81.19
Corradino et al. (2010) [46]	8	81.5	Oncologic	Scalp	143.27
Chalmers et al. (2010) [47]	2	54 ± 1	Oncologic	Scalp	144.5 ± 113.84
Messias et al. (2022) [48]	1	70	Oncologic	Scalp	150
Hughes et al. (2021) [49]	1	86	Trauma	Leg	200
Verbelen et al. (2016) [50]	1	44	Burns	Leg	200
Fung et al. (2014) [51]	1	69	Oncologic	Scalp	256

**Table 4:** Surgical techniques and methods used in IDRT-based reconstruction. All values are reported as mean ± SD where possible. In cases where multiple patients are included in the study, the number of patients experiencing the descriptor is included in parentheses. NPWT—negative pressure wound treatment.

Reference	Defect Size (cm^2^)	NPWT on Integra	NPWT on Skin Graft	Two-Stage Procedure	Skin Graft Type	Days to Grafting	Bone Preparation	Integra Meshing
Smock et al. (2010) [21]	1	No	No	Yes	STSG	21	None	No
Iino et al. (2017) [55]	1.5	No	No	Yes	PSVNSG	30	None	No
Weigert et al. (2011) [22]	2 ± 1	Yes (1), No (1)	No	Yes	STSG	26	Debridement	No
Moberly et al. (2014) [23]	2.5	No		No			None	No
Choughri et al. (2020) [9]	3.25 ± 0.96	Yes	No	Yes	STSG	27.25 ± 4.57	Debridement	No
Reynolds et al. (2018) [24]	3.67 ± 2.08	No	No	Yes	FTSG (1), STSG (2)	10 ± 3.60	None	No
Prystowsky et al. (2001) [25]	6.7	No		No			Decortication	No
Lee et al. (2008) [26]	7 ± 1.41	No	No	Yes	STSG	24.5 ± 4.95	Debridement (1), Decortication (1)	No
Ribeiro et al. (2018) [54]	8.5	No	No	Yes	STSG	21	Decortication	No
Pu et al. (2009) [27]	10	Yes	No	Yes	STSG	21	Debridement	No
Pagnini et al. (2009) [28]	12	Yes	No	Yes	STSG	14	None	Yes
Lee et al. (2024) [53]	18.62	Yes		No			None	No
Mogedas-Vegara et al. (2021) [29]	22.03 ± 2.84	No	No	Yes (50), No (16)	STSG	30.6	Burring	No
Silverstein et al. (2006) [30]	25	No	Yes	Yes	STSG	42	Decortication	No
Singh et al. (2016) [31]	27	No	No	Yes	FTSG	28	Burring	No
Yeong et al. (2006) [32]	30	No	No	Yes	STSG	21	Decortication	No
Ameloot et al. (2019) [33]	32	No	No	Yes	STSG	28	None	No
Ozgonul et al. (2018) [34]	32.47	No		No			None	No
Ćertić et al. (2023) [52]	35	No	No	Yes	STSG	18	Decortication	No
Monjanel et al. (2019) [35]	35.23 ± 8.70	No		No			None	No
Patel et al. (2022) [36]	42	No		No			Burring	No
Gonyon et al. (2003) [37]	42.37 ±53.22	No	No	Yes	STSG	21	Burring	No
Helgeson et al. (2007) [10]	45 ± 11.18	Yes	No	Yes (2), No (3)	STSG	16.5 ± 3.53	Debridement	Yes
Sartore et al. (2015) [38]	52	No	No	Yes	STSG	42	Decortication	No
Schiavon et al. (2016) [39]	56.18 ± 0.53	No	No	Yes	STSG	30.18 ± 0.53	Burring	No
Koenen et al. (2008) [40]	62.08 ± 36.06	No	No	Yes	STSG	28.61 ± 4.31	Decortication	No
Nakhi et al. (2018) [8]	71 ± 35.43	Yes	Yes	Yes	STSG	24 ± 2.83	Burring	No
Bernstein et al. (2020) [41]	74	Yes (7), No (7)	No	Yes	STSG	17.43 ± 5.34	Burring	Yes
Yeong et al. (2012) [42]	77.5 ± 82.06	No	No	Yes	STSG	18	Burring	No
Fraccalvieri et al. (2012) [16]	80 ± 84	Yes	Yes	Yes	STSG	14	Decortication	Yes
Komorowska-Timek et al. (2005) [7]	92.25 ± 43.21	No	No	Yes	STSG	48.25 ± 15.11	Burring	No
Vithlani et al. (2017) [43]	96.7	No		No			Burring	Yes
Vegesack et al. (2015) [44]	100	Yes	No	Yes	STSG	21	Burring	No
Souéid et al. (2013) [45]	126 ± 81.19	No	No	Yes	STSG	22.4 ± 3.13	Decortication (2), Burring (3)	No
Corradino et al. (2010) [46]	143.27	No	No	Yes	STSG	21	Decortication	Yes (2), No (6)
Chalmers et al. (2010) [47]	144.5 ± 113.84	Yes (1), No (1)	No	Yes	STSG	20 ± 8.45	Burring	No
Messias et al. (2022) [48]	150	Yes	Yes	Yes	STSG	30	Decortication	No
Hughes et al. (2021) [49]	200	No	No	Yes	STSG	21	Burring	Yes
Verbelen et al. (2016) [50]	200	Yes		No			Burring	No
Fung et al. (2014) [51]	256	No	No	Yes	STSG	35	Burring	No

**Table 5:** Outcomes and complications of IDRT-based reconstruction. All values are reported as mean ± SD where possible. In cases where multiple patients are included in the study, the number of patients experiencing the descriptor is included in parentheses. IDRT—Integra Dermal Regeneration Template, SG—skin graft.

Reference	Defect Size	IDRT Success Rate (%)	SG Success Rate (%)	Complications	Complication Details
Smock et al. (2010) [21]	1	100	100	No	
Iino et al. (2017) [55]	1.5	100	100	No	
Weigert et al. (2011) [22]	2 ± 1	100	100	No	
Moberly et al. (2014) [23]	2.5	100		No	
Choughri et al. (2020) [9]	3.25 ± 0.96	100	100	No	
Reynolds et al. (2018) [24]	3.67 ± 2.08	100	98.67	No	
Prystowsky et al. (2001) [25]	6.7	0		Yes	Primary Integra failure
Lee et al. (2008) [26]	7 ± 1.41	100	100	Yes (1), No (1)	Partial wound breakdown
Ribeiro et al. (2018) [54]	8.5	100	100	No	
Pu et al. (2009) [27]	10	100	100	No	
Pagnini et al. (2009) [28]	12	99	95	No	
Lee et al. (2024) [53]	18.62	0		Yes	Primary Integra failure
Mogedas-Vegara et al. (2021) [29]	22.03 ± 2.84	74.49	100	Yes (13), No (50)	Infection
Silverstein et al. (2006) [30]	25	100	80	Yes	Partial skin breakdown
Singh et al. (2016) [31]	27	100	100	No	
Yeong et al. (2006) [32]	30	100	100	No	
Ameloot et al. (2019) [33]	32	100	100	No	
Ozgonul et al. (2018) [34]	32.47	100		No	
Ćertić et al. (2023) [52]	35	100	100	No	
Monjanel et al. (2019) [35]	35.23 ± 8.70	100		Yes (2), No (8)	Ethmoidal fistula (2), Socket infection (1)
Patel et al. (2022) [36]	42	100		No	
Gonyon et al. (2003) [37]	42.37 ± 53.22	100	100	Yes (1), no (1)	Ectropion
Helgeson et al. (2007) [10]	45 ± 11.18	100	100	Yes (3). no (2)	Primary Integra failure
Sartore et al. (2015) [38]	52	100	100	No	
Schiavon et al. (2016) [39]	56.18 ± 0.53	100	100	No	
Koenen et al. (2008) [40]	62.08 ± 36.06	100	100	Yes (1), no (13)	Infection
Nakhi et al. (2018) [8]	71 ± 35.43	100	97	No	
Bernstein et al. (2020) [41]	74	92	96.54	Yes (2), no (12)	Hematoma (1), STSG failure (1)
Yeong et al. (2012) [42]	77.5 ± 82.06	87.5	87.5	Yes (2), no (2)	Partial neodermis loss (1), Partial STSG loss (1)
Fraccalvieri et al. (2012) [16]	80 ± 84	97.86	98.57	Yes (2), no (4)	Partial neodermis loss
Komorowska-Timek et al. (2005) [7]	92.25 ± 43.21	100	100	Yes (2), no (2)	Partial neodermis breakdown (1), Local tumor recurrence (1)
Vithlani et al. (2017) [43]	96.7	95		Yes	Infection
Vegesack et al. (2015) [44]	100	80	99	Yes	Infection
Souéid et al. (2013) [45]	126 ± 81.19	100	94	No	
Corradino et al. (2010) [46]	143.27	100	100	No	
Chalmers et al. (2010) [47]	144.5 ± 113.84	100	100	No	
Messias et al. (2022) [48]	150	70	85	Yes	Partial skin breakdown
Hughes et al. (2021) [49]	200	100	100	No	
Verbelen et al. (2016) [50]	200	0		Yes	Integra failure
Fung et al. (2014) [51]	256	100	100	Yes	Hematoma

## Data Availability

Not available.
